# Genome editing of tumor specific T cells for sustained functionality in a suppressive tumor microenvironment

**DOI:** 10.1186/2051-1426-3-S2-P43

**Published:** 2015-11-04

**Authors:** Sara R Pedersen, Per Thor Straten

**Affiliations:** 1 Center for Cancer Immune Therapy, Dept. of Hematology, Copenhagen University Hospital, Herlev, Denmark, Herlev, Denmark; 2 Centre for Cancer Immune Therapy (CCIT), Copenhagen University Hospital Herlev, Herlev, Denmark, Herlev, Denmark

## Background

Solid tumors are known to be infiltrated by cells of the immune system, and a positive prognostic value of tumor infiltrating CD8 T cells are found in most cancers. Such tumor infiltrating lymphocytes (TIL) can be expanded by *in vitro* culture and used for adoptive cell transfer (ACT) in the treatment of metastatic melanoma. This strategy has led to clinical responses in about 50% of patients with potential cures in 20%, and Center for Cancer Immune Therapy has launched a randomized Phase III trial using this approach. Although this is an impressive breakthrough in the treatment of metastatic melanoma, 80% of patients still succumb to disease, and 50% have no benefit from treatment. Thus, TILs are quite often unable to fully control and eliminate the cancer, and inappropriate regulation of immune mechanisms is thought to play a role in this. A number of tumor suppressor mechanisms have already been described, including tumors inducing intrinsic inhibitory pathways in TILs. This renders TILs less active and tumors can hereby avoid immune recognition and destruction.

## Methods

In the current project, we want to genetically engineer T cells to block inhibitory pathways prior to ACT. Taking advantage of the recently developed CRISPR/Cas9 technology, genes associated with inhibitory pathways will be deleted from the genome of tumor specific T cells. These T cells will then be tested for *in vivo* functionality in the tumor microenvironment using a humanized mouse model. We believe that success in genetically engineering T cells to elicit sustained functionality in a suppressive tumor microenvironment would be of great importance for the further development of ACT.

